# Two Weeks of Mirror Exposure Enhances Sensorimotor Cortex Activation but not Facial Mimicry in 4‐Month‐old Infants

**DOI:** 10.1111/desc.70221

**Published:** 2026-05-10

**Authors:** Chloe Richmond, Lucy Parrett, Elizabeth Tye, Carina C. J. M. de Klerk

**Affiliations:** ^1^ Centre for Brain Science, Department of Psychology University of Essex Colchester UK

**Keywords:** associative learning, EEG, EMG, facial mimicry, infancy, sensorimotor experience

## Abstract

This study aimed to test the causal role of sensorimotor experience in the development of facial mimicry. We systematically manipulated 4‐month‐old infants’ experience with their own facial actions, and measured the effect on their sensorimotor cortex activation and facial mimicry when they observed others’ facial actions. Infants in the mirror condition received two weeks of daily sensorimotor experience with their own facial actions via a toy mirror, while infants in the control condition played with the same toy without the mirror for the same amount of time. Before and after this experience, we measured infants’ facial mimicry using electromyography (EMG) and their sensorimotor cortex activation using electroencephalography (EEG) while they observed videos of other infants’ facial actions. As predicted, infants in the mirror condition showed a greater increase in sensorimotor cortex activation during the observation of other infants’ facial actions than infants in the control condition. However, this greater neural activation did not translate into a greater increase in facial mimicry in the mirror group. These findings suggest that although the neural processing of others’ facial actions was enhanced as a result of the sensorimotor training, longer training periods may be necessary for this to lead to greater facial mimicry.

**Summary:**

- We investigated the role of sensorimotor experience in infant facial mimicry by assigning infants to either a mirror or control condition
- Infants in the mirror condition received two weeks of daily mirror exposure
- Mirror training enhanced sensorimotor cortex activity during the observation of facial actions
- No corresponding increase in facial mimicry was observed

## 1 Introduction

Mimicry, the spontaneous tendency to copy others’ actions, is thought to play an important role in facilitating social bonds. For example, it enhances liking and rapport between strangers (Chartrand and Bargh 1999) and increases helpfulness (Van Baaren et al. 2004). Facial mimicry is of particular significance because it relates to empathy (see Holland et al. 2021 for a meta‐analysis) and is thought to enhance our ability to recognise others’ emotions (Borgomaneri et al. 2020; Lydon and Nixon 2014; Stel and van Knippenberg 2008). However, despite the important functions facial mimicry serves in supporting social rapport and empathy, there is still significant debate surrounding its development.

Facial mimicry is thought to be supported by the mirror neuron system (MNS)—a set of brain areas that are activated both when we perform and when we observe actions (e.g. Wang et al. 2011). Thus seeing someone's facial actions is thought to activate a similar motor representation in the observer, resulting in subtle activation of the corresponding facial muscles. Indeed, it has been shown that activation of motor areas during the observation of facial expressions correlates with facial mimicry responses as measured by facial electromyography (EMG) (Likowski et al. 2012). One of the most popular views regarding the ontogeny of the MNS is that this coupling between “seeing” and “doing” is inborn (e.g. Lepage and Théoret, 2007; Meltzoff and Decety, 2003) and that it supports infants’ ability to copy others’ actions from birth (e.g. Simpson et al., 2014). However, reports of neonatal imitation have been subject to extensive debate (e.g. Jones, 2009; Meltzoff et al. 2018; Oostenbroek et al. 2016, Oostenbroek et al. 2018). For example, although several studies have failed to find evidence for neonatal imitation (e.g. Hayes and Watson 1981; Koepke et al. 1983; McKenzie and Over 1983; Oostenbroek et al. 2016), some have argued that these non‐replications reflect procedural differences or methodological limitations (e.g. Meltzoff and Moore 1983). In addition, another line of research suggests that tongue protrusion—the facial action that is most reliably reproduced by neonates—may reflect a general oral exploratory response that occurs in response to any stimuli that arouse or interest infants rather than imitation per se (Jones 1996, 2006). Consequently, the debate regarding when facial mimicry first occurs is still ongoing, with an upcoming ManyBabies study (see https://manybabies.org/MB6/) aiming to provide a large‐scale multi‐lab conceptual replication of the original neonatal imitation work using standardised methods.

An alternative perspective regarding the ontogeny of the MNS is that, rather than being inborn, couplings between visual and motor representations of actions instead develop as a result of associative learning during correlated sensorimotor experience (Heyes 2001; Keysers and Perrett 2004). These accounts propose that through repeated experience of seeing and doing the same action (e.g. when someone copies the infant's facial expressions), infants form links between the action's sensory and motor representations causing the motor representation to become activated in response to the mere observation of an action that is similar (e.g. when the infant sees someone else's facial expression) (Ray and Heyes 2011). Thus instead of being supported by inborn couplings between seeing and doing, it is thought that facial mimicry needs to be learned. Support for these accounts has been provided by studies demonstrating that in adults, correlated sensorimotor experience can enhance, abolish, reverse, or induce perceptual–motor couplings (e.g. see Cook et al. 2014 for a review). If perceptual‐motor couplings are required for mimicry, and if these couplings indeed develop through associative learning, then the extent to which an observed action elicits mimicry should depend on the extent to which the infant has experienced repeated sensorimotor contingency with this action. For perceptually opaque actions, such as facial actions, that infants cannot observe themselves perform, visual feedback from imitative social partners is thought to provide the necessary input to form the perceptual‐motor couplings that support mimicry (Ray and Heyes 2011). Work by Rayson et al. (2017) has provided evidence for this idea by showing that maternal imitation of infants’ facial expressions at 2 months was positively associated with infants’ motor cortex activation—as measured by alpha suppression over the sensorimotor cortex—during the observation of facial expressions at 9 months. Additionally, in our previous work, we found that mothers’ tendency to imitate their 4‐month‐olds’ facial expressions was positively associated with the infants’ facial mimicry (de Klerk et al. 2019). Together, these studies are consistent with the idea that facial mimicry is supported by perceptual‐motor couplings that are formed through correlated sensorimotor experience. However, several important questions remain unanswered. First, the previous research is correlational and therefore it is possible that the relationship between maternal imitation and infant action processing and mimicry reflects a more general enhanced prosocial attitude or a greater attention to others’ actions driven by heritable factors. Second, while it is assumed that motor cortex activation during action observation reflects the existence of perceptual‐motor couplings that give rise to facial mimicry, no study to date has measured both motor cortex activation and facial mimicry in the same infants. Given the important functions facial mimicry serves in supporting successful social interactions and in the mirroring and recognising others’ emotion expressions, elucidating the mechanisms underlying its development is crucial for our understanding of both the typical and atypical development of social cognition.

The current preregistered study (https://tinyurl.com/59d6m95v) aimed to test the causal role of sensorimotor experience in the development of facial mimicry by systematically manipulating 4‐month‐olds’ experience with their own facial actions, and measuring the effect on their motor cortex activation and facial mimicry when observing others’ facial actions. We focused on 4‐month‐old infants because this is the earliest age at which facial mimicry as measured by facial EMG has been demonstrated (e.g. de Klerk et al. 2018; Isomura and Nakano 2016). We selected this early age range to maximise the likelihood that manipulating sensorimotor experience would influence developing sensorimotor representations of facial actions. Infants in the mirror condition received two weeks of daily sensorimotor experience with their own facial actions via a toy mirror, while infants in the control condition played with the same toy without the mirror for the same amount of time. Before and after this experience, we measured infants’ facial mimicry using EMG and their sensorimotor alpha suppression (4‐7 Hz) using electroencephalography (EEG) while they observed videos of other infants’ facial actions. This approach provided a controlled and replicable way to manipulate infants’ sensorimotor experience, while ensuring a close alignment between the training and test stimuli, thereby allowing a direct test of associative learning mechanisms. We focused on sensorimotor alpha suppression in the 4–7 Hz range because previous research has shown that this frequency band reflects the infant analogue to the adult sensorimotor alpha rhythm, with decreases in power in this frequency range reflecting activation of the sensorimotor cortex in infants this age (Berchicci et al. 2011). We hypothesised that infants in the mirror condition would show a greater increase in facial mimicry and sensorimotor cortex activation during the observation of others’ facial actions than infants in the control condition. We also expected that the amount of mirror exposure during the two‐week training period would predict the change in facial mimicry and sensorimotor cortex activation between pre‐ and post‐test.

## 2 Methods

### 2.1 Participants

For the EEG analyses, the final sample consisted of 52 infants (*N* = 26 in the mirror condition and *N* = 26 in the control condition) aged between 3.5 and 4.5‐months‐old (29 females, mean age at pre‐test = 122.25 days, SD = 11.14). An additional 46 infants were tested but excluded because they did not provide sufficient artifact‐free trials at post‐test (*N* = 42) or because they did not complete the training period (*N* = 4).

For the EMG analyses, the final sample consisted of 76 infants (*N* = 37 in the mirror condition and *N* = 39 in the control condition) (44 females, mean age at pre‐test = 122.25, SD = 11.25). An additional 22 infants were tested but excluded because they did not provide sufficient trials at post‐test (*N* = 18), or because they did not complete the training period (*N* = 4).

All infants were born healthy and with normal birth weight. Participants were recruited through the Essex Babylab database which contains details of caregivers who have registered interest in participating in child development research with their infant. Caregivers were recruited to sign up to this database by researchers attending baby classes, leaflets in local centres/libraries, and through posts shared via social media. Caregivers were recruited from the Colchester area, a predominantly urban and suburban area in the southeast of England, characterised by a majority White British population and a mix of middle‐ and working‐class communities. In line with the GDPR's data minimization principle and guidance from our institutional ethics committee, information on participants’ ethnicity and socioeconomic status was not collected, as it was not essential to the aims of the study. Prior to participation, caregivers provided informed consent via an online form on Qualtrics. Participants received a £5 Amazon voucher and a baby grow to thank them for their participation.

### 2.2 Design and Procedure

This study used a pre‐test, training, post‐test design to investigate the effect of sensorimotor experience with their own facial actions on infants’ sensorimotor cortex activation and facial mimicry in response to video stimuli of other infants’ facial actions. Infants were randomly allocated to one of the two conditions for the training phase of the study: mirror or control. Infants assigned to the mirror condition received a Taf Toys tummy time book containing a 14×10 cm infant‐friendly mirror. Infants assigned to the control condition received the same toy, but access to the mirror was restricted using Velcro. Caregivers were asked to place their infant in front of the toy for approximately 20 minutes per day over a 14 day period, which could be divided into multiple shorter sessions across the day. Caregivers of infants in the mirror condition were instructed to ensure that the infant was facing the mirror, but that they could use the other pages of the book to maintain the infant's interest (for example, by playing peek‐a‐boo with the mirror or crinkling the textured pages). Caregivers of infants in the control condition were also encouraged to use the pages of the book to keep the infant engaged, and were asked, where possible, to restrict access to mirrors in the home during the training period. Caregivers were asked to complete an online questionnaire via Qualtrics for each day of the training phase. Embedded within the questionnaire were the following questions of interest: “How many times did your baby play with their new toy today?”, “How many minutes (in total) did your baby spend playing with their new toy today?”, and “How many minutes (in total) did your baby spend looking at their own reflection”?

### 2.3 Pre‐ and Post‐Test

**FIGURE 1:**
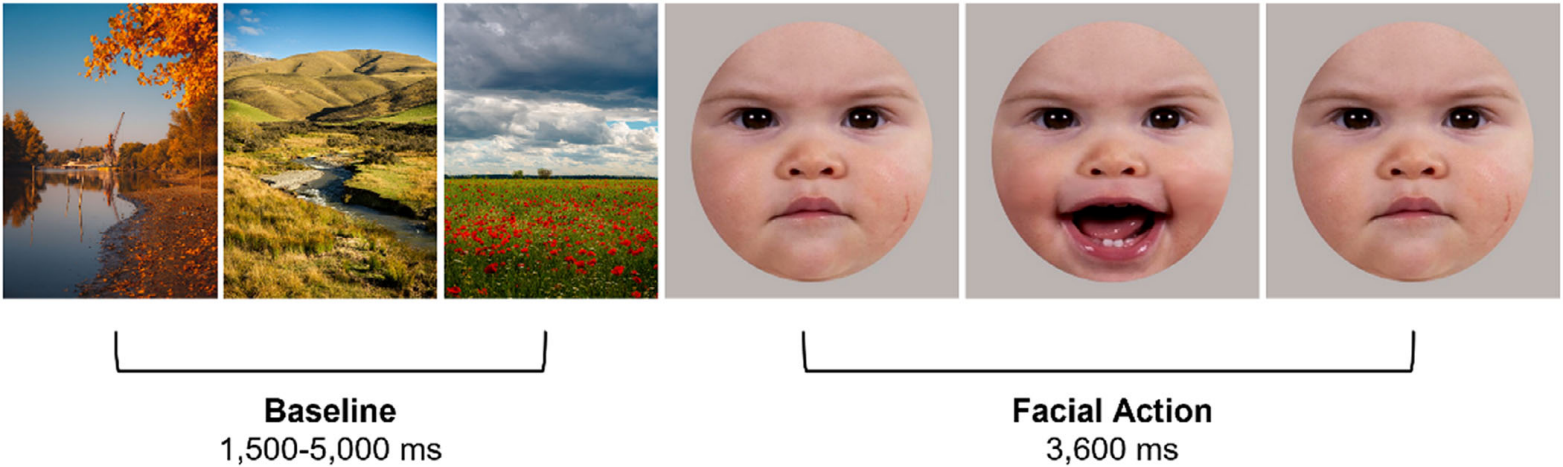
Example of a trial. *Note*: Schematic overview of the stimulus presentation including a baseline stimulus and a facial action video clip.

The experiment took place in a dimly lit room, with the infant sitting on their caregiver's lap approximately 50‐cm from a 60‐cm computer screen. Infants were presented with videos of infants displaying facial expressions. There were two trial types: Eyebrow and Mouth actions. We created five videos for each trial type resulting in a total of 10 stimulus videos. To create these stimuli, images of eight different Caucasian infants with the most distinct changes in the eyebrow and mouth region were selected from the Tromso Infant Faces Database (Maack et al. 2017). The model infants included in the mouth action stimuli ranged from 4–12 months (mean age ≈ 8.2 months), while those selected for the eyebrow action stimuli ranged from 6–12 months (mean age ≈ 8.8 months). Some model infants contributed images to both trial types, whereas others were used for only one trial type. For mouth actions, the selected images were previously rated as depicting predominantly happiness, while for the eyebrow actions the images were previously rated as depicting a blend of negative emotions, such as surprise and fear or sadness, disgust, and anger (see Maack et al. 2017 for stimulus validation). Importantly, all selected images depicted clear, prototypical facial actions that are within the motor repertoire of 4‐month‐old infants (Sullivan and Lewis 2003), ensuring that our stimuli were appropriate for the participant age group. Each stimulus was created by first generating an ‘end‐state’ image in which an image of the infant with a neutral facial expression was combined with an image of the same infant with an emotional facial expression that involved either the eyebrow or mouth region. This ensured that only the relevant part of the face (e.g. eyebrows or mouth) appeared to be moving in the final videos. For the Eyebrow action stimuli we merged the bottom half of the neutral face images with the top half of the eyebrow movement face images. For the Mouth action stimuli we merged the bottom half of the mouth movement face images with the top half of the neutral face images. GIMP software was used to blur the ‘join’ of these faces to create seamless, lifelike images of infant faces. Using Microsoft PowerPoint, we placed an oval cut‐out of the infant faces onto a grey background, to remove any distracting features, such as clothing or hair. Using these images, we then created video clips in FantaMorph that transitioned from the fully neutral facial expression image to the end‐state image and then back to the neutral expression again (see Figure 1). This morphing process created dynamic, natural‐looking facial movements within each 3.6 second video. Pilot testing with adult participants (*N* = 9) confirmed that the selected images elicited facial mimicry in adults.

The 3.6 second face videos were presented in a random order, alternated with baseline stimuli consisting of three static pictures of landscapes with a random duration between 1500 and 5000 ms to allow for any mimicry responses to subside before the next video was presented (see Figure 1). If necessary, attention‐grabbing sounds were played to draw the infants’ attention back to the screen. Videos were presented for approximately 50 trials or until the infant's attention could no longer be attracted to the screen. Infants were video‐recorded in OBS Studio via a high‐definition webcam situated below the computer screen. The stimulus presentation screen was simultaneously recorded in OBS. The resulting side‐by‐side videos (3840×1080 resolution, 60 fps) provided sufficient detail to allow for offline video coding. Because the coders were involved in some of the testing sessions, full blinding to condition and session was not possible. However, as the coding scheme was objective and based on clearly defined behaviours, we believe the likelihood of bias was minimised.

### 2.4 Recording and Processing of EEG

EEG data was recorded using a 124‐channel geodesic sensor net (EGI Inc) and NetStation version 5.4.2 with a sampling rate of 500 Hz. EEG was recorded with respect to the vertex electrode with a hardware filter between 0.1 Hz (high‐pass) and 100 Hz (low‐pass).

Each EEG session was video‐coded to exclude any trials in which the infant did not attend to the screen for at least 3000 ms or made any hand or arm movements. Trials were also excluded if the infant was inattentive or moving their arms or hands during the baseline period. The data was pre‐processed in NetStation, and filtered between 0.3 Hz (high‐pass) and 40 Hz (low‐pass) and segmented into 5400 ms trials, including a: 1000 ms baseline period, a 3600 ms analysis period, and a 400 ms buffer on either side of the segment. Each segment was visually inspected to manually mark artefacts and channels with poor signal quality. If a trial had more than 30^1^ channels marked as bad, it was excluded. If fewer than 30 channels were marked as bad, the bad channels were replaced with data interpolated from surrounding, non‐marked channels. Only infants with at least 8 artifact‐free trials at post‐test were included in the analyses^2^. Infants contributed a mean of 16.56 (SD = 5.26) artefact‐free trials to the analysis at pre‐test, and 13.62 (SD = 6.61) at post‐test. For infants who did not provide sufficient valid data at pre‐test, but did at post‐test, their pre‐test data was imputed by replacing it with the mean pre‐test alpha suppression of their condition. Of the 52 infants included in the EEG analyses, 34 did not have sufficient valid data at pre‐test and thus their pre‐test data was imputed as per our preregistration. Due to this greater‐than‐anticipated proportion of missing pre‐test data, we conducted an additional un‐preregistered analysis focusing on the post‐test scores only, that also assessed whether there were any systematic differences between infants with versus without valid pre‐test data (see exploratory EEG analysis section below).

Time‐frequency analyses were conducted on artifact‐free trials using Wtools—a Matlab‐based toolbox for time‐frequency analysis (Ferrari et al. 2024). The data were re‐referenced to the average prior to analysis. Hereafter, Morlet wavelets were computed at 1 Hz intervals in the 3 to 20 Hz range using WTools. To eliminate distortion created by the wavelet transformation, the 400 ms buffer at the start and end of each trial was removed. Activity in the 4–7 Hz‐frequency range during the baseline period (1000–500 ms before stimulus onset) was subtracted from activity during the 3600 ms analysis period. Average wavelet coefficients were calculated for each infant and condition by taking the mean across the trials. Finally, baseline‐corrected 4–7 Hz activation over the channels of interest were averaged together.

**FIGURE 2:**
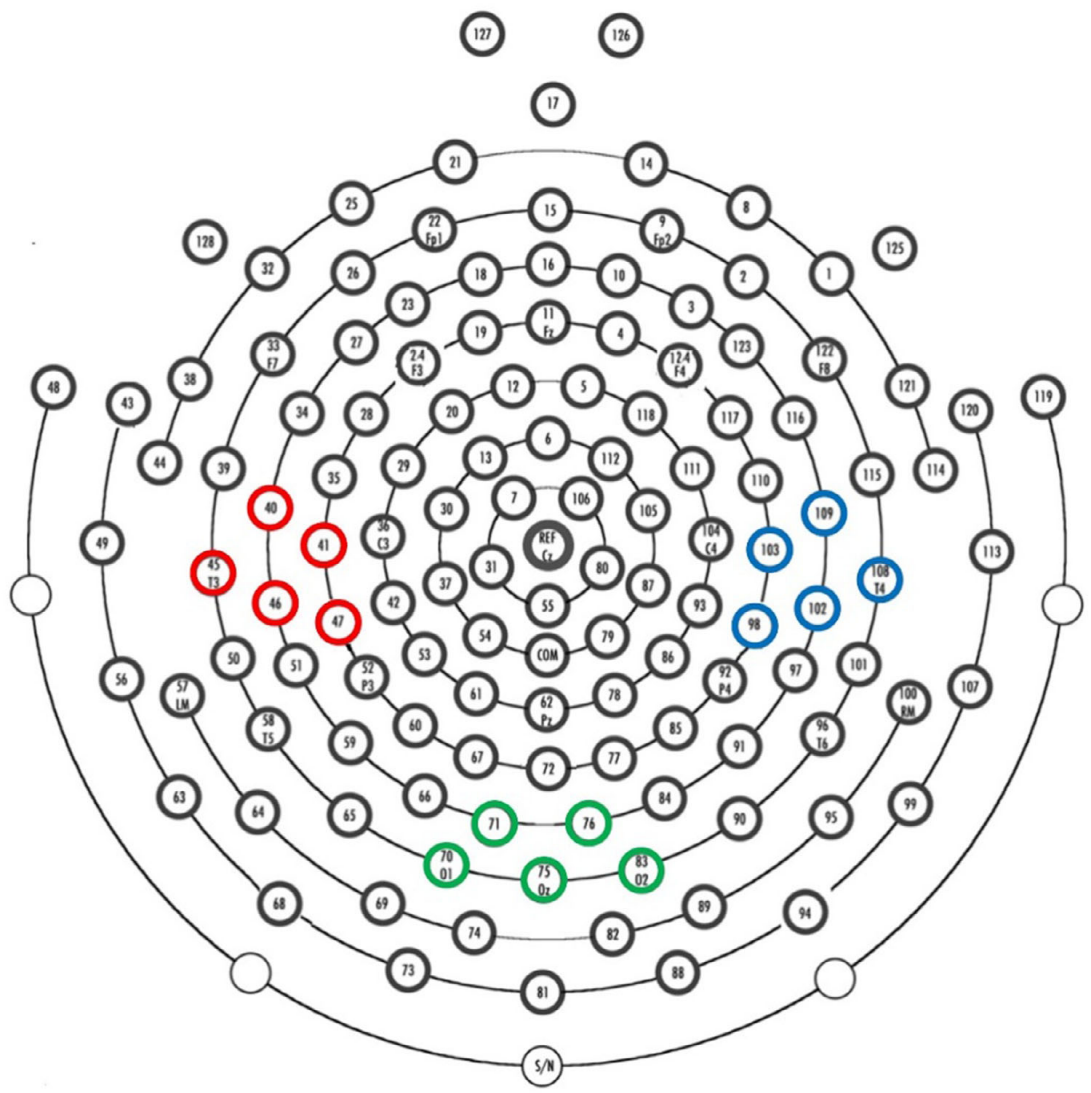
Geodesic sensor net with three channel clusters marked. *Note*: The Geodesic sensor Net 128 channel lay‐out with the three channel clusters of interest marked: left (40, 41, 45, 46, 47 in red) and right (98, 102, 103, 108, 109 in blue) face representation areas and the occipital area (70, 71, 75, 76, 83 in green).

We used a preregistered functional localisation procedure based on adult pilot data to select clusters of 5 electrodes over both the left and right sensorimotor face representation area for analysis. We asked adult participants to perform the same facial actions as those presented to the infants during the pre‐ and post‐test session, and selected the channels that showed the greatest sensorimotor alpha suppression over each hemisphere. The selected channels for the left sensorimotor face representation area were: 40, 41, 45, 46, 47. The selected channels for the right sensorimotor face representation area were: 98, 102, 103, 108, 109. These clusters of channels fall over the lateral sensorimotor cortex where the infant face representation area is expected to be located, based on the finding that sensorimotor alpha suppression is somatotopically organised in infancy (e.g. De Klerk et al. 2015; Saby et al. 2013) and on the topographic distribution of alpha suppression observed in our dataset. Data from channels 70, 71, 75, 76, and 83 overlying occipital areas were also used to confirm that any differences in alpha suppression between the two conditions were not driven by differences in visual processing. Figure 2 illustrates the channels selected for analyses.

### 2.5 Recording and Processing of EMG

The EMG recording and processing procedure was similar to that of de Klerk et al. (2018). Bipolar EMG recordings were made using paediatric surface electrodes that were placed over the zygomaticus muscle region and the corrugator muscle region with an inter‐electrode spacing of approximately 1 cm. The electrodes were connected to a Biopac Bionomadix transmitter box that amplified the electrical muscle activation, which was in turn recorded using the software AcqKnowledge at a sampling rate of 2000 Hz.

Each EMG session was video‐coded to exclude any trials in which the infant: did not attended to the screen for at least 3000 ms, vocalised, looked visibly upset (such as a pouted lip or wincing), yawned, chewed on something, had something in their mouth, startled, or pulled the wires of the EMG electrodes. Only infants with at least 5 trials per trial type were included in the analysis. On average, the included infants contributed a mean of 10.72 Eyebrow trials (SD = 4.00) and 11.79 Mouth trials (SD = 4.37) to the analysis at pre‐test, and 10.13 Eyebrow trials (SD = 4.06) and 10.66 Mouth trials (SD = 3.79) at post‐test. Of the 76 infants included in the analyses, 19 did not have a sufficient number of good trials at pre‐test, and thus their pre‐test mimicry score was imputed by replacing it with the mean mimicry scores of their condition as per our preregistration.

The EMG signal was filtered (high‐pass: 30 Hz, low‐pass: 500 Hz), smoothed (root mean square over 20 ms bins) and rectified (converted to absolute values). The EMG signal was segmented into 3600 ms epochs, and the average activity in each epoch was normalised (i.e., expressed as z‐scores) within each participant and each muscle group, before the epochs for each trial type were averaged together. This allowed for meaningful comparison of values between muscle regions, as well as reduced the impact of individual differences in reactivity on the group mean. Standardised activation over the two muscle groups was compared rather than baseline‐corrected means, as the latter would require each trial to be preceded by a valid baseline segment, which would substantially reduce the number of trials available for analysis.

Mimicry is defined as the presence of greater activation over corresponding muscles than over non‐corresponding muscles during the observation of facial actions (e.g. McIntosh et al. 2006). Similar to previous research (e.g. de Klerk, 2018), facial mimicry scores were calculated by subtracting EMG activity over the non‐corresponding muscle from EMG activity over the corresponding muscle, with a more positive score indicating greater facial mimicry. We also calculated a mimicry difference score by subtracting the facial mimicry score at pre‐test from the facial mimicry score at post‐test.

## 3 Results

### 3.1 Manipulation Check

We first performed a manipulation check to investigate compliance to the training protocol. We conducted one‐way ANOVAs on the relevant variables from the daily parental questionnaire responses. These analyses revealed the expected significant difference between the average time (in minutes) per day the infants spent looking at their reflection between the two groups, *F*(1, 75) = 42.308, *p*≤.001, with infants in the mirror group on average spending more time looking at their reflection per day (*M* = 18.42, *SD* = 9.138) than infants in the control group (*M* = 6.573, *SD* = 6.742). Additionally, there was no difference in the number of times or the duration of toy play between the two groups, all *p*‐values ≥0.153 (average around 2–3 times a day for a total of 15–17 min a day in both groups).

### 3.2 EMG Results

**FIGURE 3:**
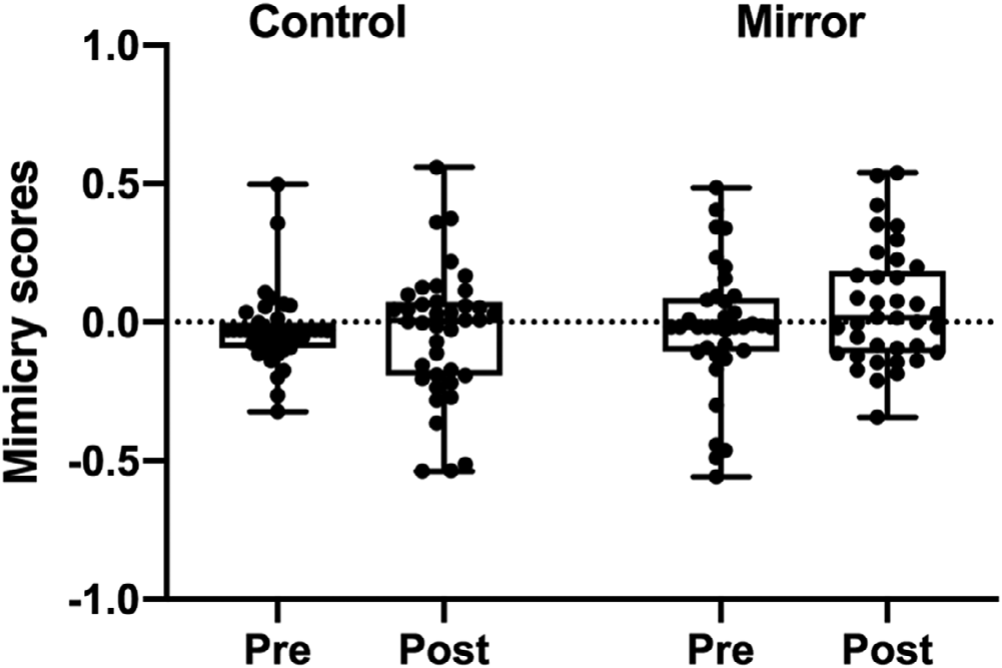
Boxplots of mean mimicry scores at pre‐ and post‐test per condition. *Note*: The whiskers represent the minimum and maximum data points, and individual data points are marked with circles. The line within the box represents the median.

Facial mimicry scores were examined using a repeated measures ANOVA with time (pre‐test vs. post‐test) as within‐subjects factor and condition (mirror vs. control) as between‐subjects factor. There was no significant effect of time, *F*(1, 74) = 0.840, *p* = 0.362, np^2^ = 0.011, and no interaction between time and condition, *F*(1, 74) = 1.406, *p* = 0.240, np^2^ = 0.019, suggesting that there was no significant change in mimicry scores between pre‐ and post‐test in either of the groups. There was also no significant main effect of condition, *F*(1,74) = 2.435, *p* = 0.123, np^2^ = 0.032. Figure 3 shows the mean mimicry scores at pre‐ and post‐test for the two conditions. Mimicry scores did not differ significantly from zero, all *p*‐values ≥ 0.065 (one‐sided). Exploratory analyses including trial type (mouth vs. eyebrow action) as a within subjects factor, showed no main effects or interactions involving trial type, indicating that the results were the same for the mouth and eyebrow actions (see Supplementary Materials).

Additional exploratory analyses on baseline‐corrected EMG activity were performed to assess whether facial mimicry might emerge when accounting for individual differences in baseline muscle activation. These analyses yielded the same pattern of null results (see Supplementary Materials). Additionally, we coded for and analysed overt (i.e. visible) facial mimicry (see Supplementary Materials) and although we found evidence of some infants in the mirror condition showing overt mimicry of mouth actions at pre‐test, there was no evidence for overt mimicry in either of the groups at post‐test.

**FIGURE 4:**
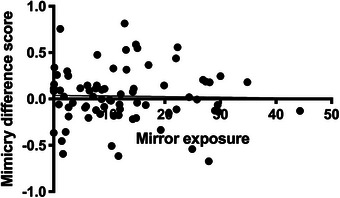
Relationship between mirror exposure and mimicry difference scores. *Note*: Scatterplot of the relationship between the average time the infant's spent looking at their reflection (in minutes per day) and the mimicry difference score across both groups.

We investigated the relationship between average mirror exposure per day and the change in facial mimicry between pre‐ and post‐test using a simple linear regression analysis. As illustrated in Figure 4, this analysis showed that the mean time infants spent looking at their reflection (in minutes per day) did not predict the change in facial mimicry scores, *B* = ‐0.001, *SE* = 0.003, *t* = ‐0.195, *p* = 0.846.

### 3.3 EEG Results

Sensorimotor alpha suppression was examined using a repeated measures ANOVA with time (pre‐test vs. post‐test) and scalp location (left sensorimotor face area, right sensorimotor face area, and occipital cortex) as within‐subjects factors and condition (mirror vs. control) as a between‐subjects factor. There was a significant interaction between location and condition, *F*(2, 100) = 6.281, *p* = 0.003, np^2^ = 0.112, and a marginally significant interaction between time, location, and condition, *F*(2, 100) = 3.047, *p* = 0.052, np^2^ = 0.057. There were no other significant main effects or interactions, all *p*‐values ≥ 0.076. Follow‐up comparisons indicated that the interaction between location and condition was driven primarily by greater alpha suppression in the Mirror compared to the control group over the right sensorimotor face area, *p* < 0.001 (Bonferroni corrected), whereas no group differences were found over the left sensorimotor or occipital area (all *p*‐values ≥ 0.220).

**FIGURE 5:**
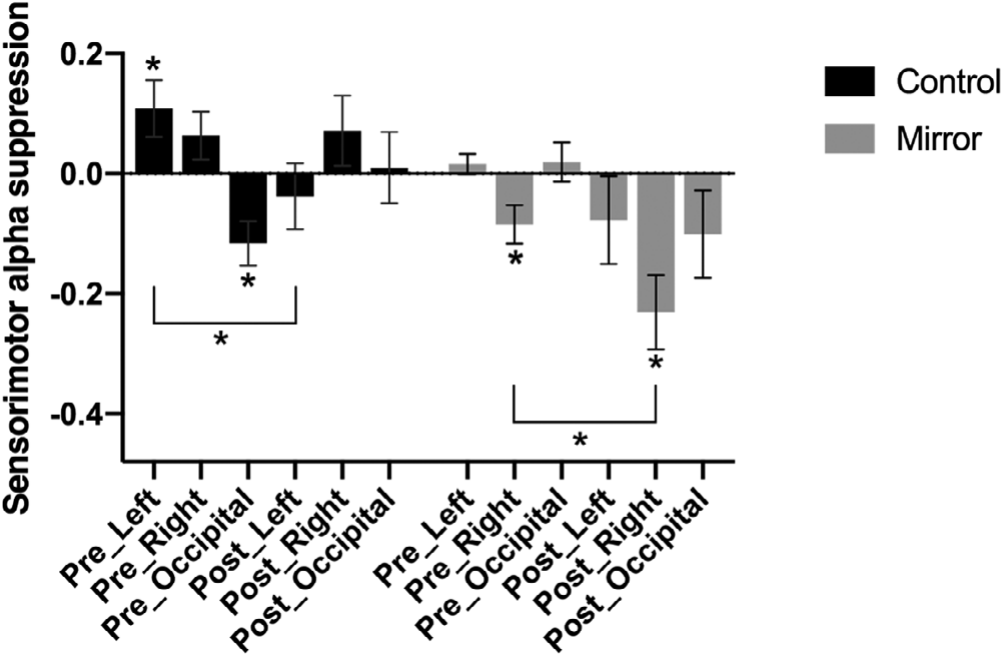
Mean alpha suppression across the three scalp locations at pre‐ and post‐test for the mirror and control condition. *Note*: This figure shows the alpha suppression across the three scalp locations (left sensorimotor cortex, right sensorimotor cortex, and occipital lobe) at both pre‐ and post‐test for the two groups. Error bars indicate the standard error of the mean (SEM). *p < 0.05.

To follow up on the marginally significant three‐way interaction, separate repeated measures ANOVAS with time (pre‐test vs. post‐test) and scalp location (left sensorimotor face area, right sensorimotor face area, and occipital cortex) as within‐subjects factors were conducted for each condition. These analyses showed that in the Control condition there was a significant main effect of location, *F*(2, 50) = 3.908, *p* = 0.026, np^2^ = 0.135, and a significant interaction between time and location, *F*(2, 50) = 5.518, *p* = 0.007, np^2^ = 0.181. The significant main effect of location in this condition was driven by the fact that overall, there was greater alpha suppression over the occipital area than over the sensorimotor face areas (average left sensorimotor alpha suppression = 0.035, right sensorimotor alpha suppression M = 0.067, occipital alpha suppression = ‐0.053). However, none of the pairwise differences between channel locations remained significant after Bonferroni correction, all *p*‐values ≥ 0.062. As illustrated in Figure 5, the significant interaction between time and location in this group was driven by the fact that there was a non‐significant decrease in alpha suppression over the right sensorimotor face area and the occipital area between pre‐ and post‐test (all *p*‐values ≥ 0.101), while there was an increase in suppression between pre‐test and post‐test over the left sensorimotor face area, *p* = 0.044 (Bonferroni corrected). This apparent change is attributable to the relatively high alpha power at pre‐test rather than a meaningful increase in alpha suppression at post‐test, as alpha suppression over the left sensorimotor cortex in this group was not significantly different from zero at post‐test, *t*(25) = ‐0.690, *p* = 0.248.

In the Mirror condition, there was a significant main effect of location, *F*(2, 50) = 3.725, *p* = 0.031, np^2^ = 0.130, and a significant main effect of time, *F*(1, 25) = 7.213, *p* = 0.013, np^2^ = 0.224. The significant main effect of location in this condition was driven by the fact that overall, there was greater alpha suppression over the right sensorimotor face area compared to the other two areas (mean left sensorimotor alpha suppression = ‐0.031, mean right sensorimotor alpha suppression M = ‐0.158, mean occipital alpha suppression = ‐0.040). None of the differences were significant after Bonferroni correction, all *p*‐values ≥ 0.072. The significant main effect of time was driven by the fact that overall, there was greater alpha suppression at post‐test than at pre‐test across scalp locations in this group, *p* = 0.013 (Bonferroni corrected). Although this increase was observed across scalp locations, this effect was most pronounced over the right sensorimotor area, where the difference between pre‐ and post‐test was significant, *p =* 0.010 (Bonferroni corrected) and the alpha suppression at post‐test was significantly different from zero, *t*(25) = ‐3.722, *p* = 0.001.

### 3.4 Exploratory EEG Analysis

**FIGURE 6:**
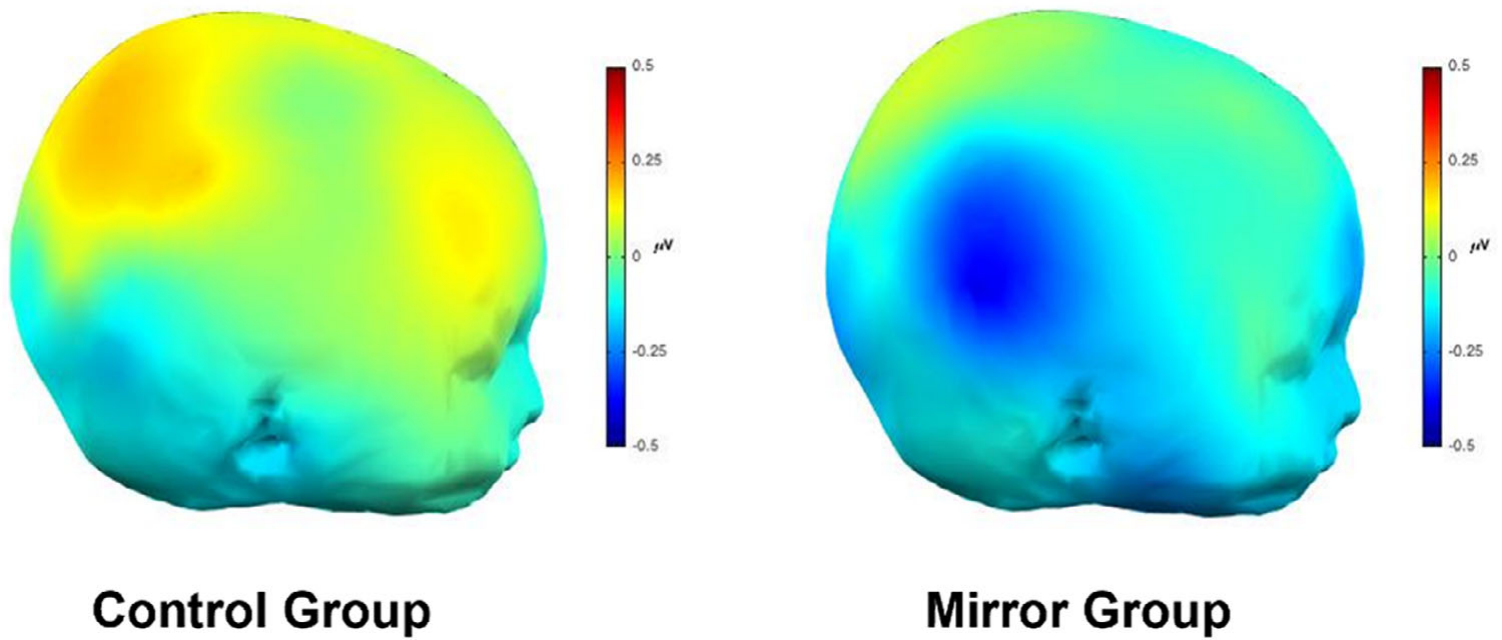
Sensorimotor alpha suppression at post‐test. *Note*: Scalp plots illustrating the sensorimotor alpha suppression at post‐test over the right hemisphere in response to the facial action stimuli in the two conditions.

Because of the large proportion of missing pre‐test data, we conducted an additional un‐preregistered MANOVA with post‐test alpha suppression at the three scalp locations (left sensorimotor face area, right sensorimotor face area, and occipital cortex) as dependent variables and condition (mirror vs. control) and pre‐test data status (observed versus imputed) as between subjects factors. The multivariate test indicated a significant effect of condition, Wilks’ *λ* = 0.807, *F*(3, 46) = 3.664, *p* = 0.019, *np^2^* = 0.193. There was no significant effect of pre‐test data status, Wilks’ *λ* = 0.973, *F*(3,46) = 0.419, *p* = 0.740, *np^2^* = 0.027, and no condition by pre‐test data status interaction, Wilks’ *λ* = 0.977, *F*(3,46) = 0.369, *p* = 0.776, *np^2^* = 0.023. These findings indicate that post‐test data did not differ systematically between infants with imputed versus observed pre‐test data. Follow‐up univariate tests demonstrated that the significant effect of condition was driven by differences over the right sensorimotor face area, *F*(1,48) = 10.451, *p* = 0.002, *np^2^* = 0.179. Condition effects over the other scalp locations were not significant, all *p*‐values ≥ 0.206. As illustrated in Figure 6, infants in the mirror group showed significantly greater alpha suppression over the right sensorimotor face area at post‐test compared to those in the control group.

**FIGURE 7:**
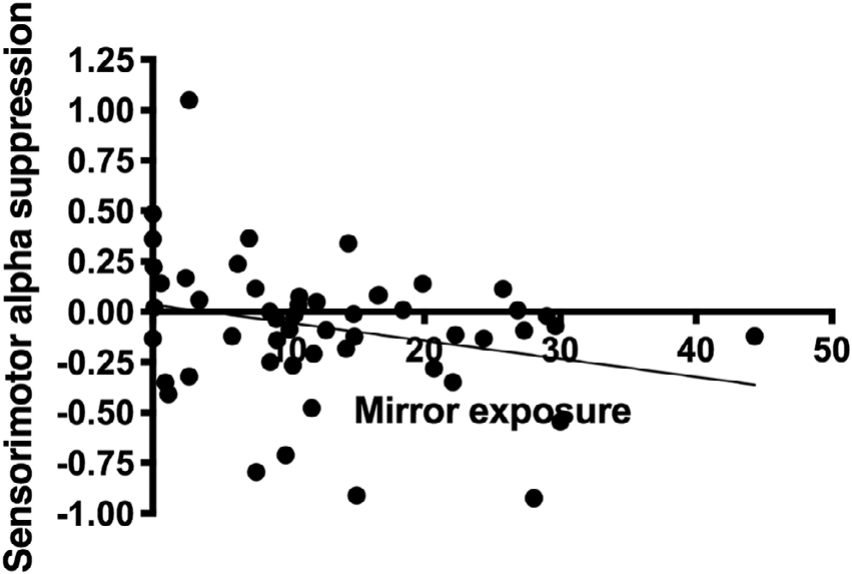
Correlation between mean time looking at reflection and sensorimotor alpha suppression over the right face area at post‐test. *Note*: This figure illustrates the relationship between the infants’ mean time spent looking at their reflection per day during the training period and their sensorimotor alpha suppression over the right face area at post‐test across both groups.

As per our preregistration, we investigated the relationship between average mirror exposure per day and the change in sensorimotor alpha suppression over the face areas between pre‐ and post‐test using simple linear regression analyses. These analyses did not show a significant relationship between mirror exposure and the change in sensorimotor cortex activation, all *p*‐values ≥ 0.195. However, given that a large proportion of the pre‐test scores was imputed, we also performed an additional exploratory analysis to investigate the relationship between mirror exposure and mean sensorimotor alpha suppression over the right face area at post‐test. As illustrated in Figure 7, this relationship was marginally significant, *B* = ‐0.009, *SE* = 0.005, *t* = ‐1.924, *p* = 0.060, which aligns with the expected trend that sensorimotor alpha suppression should increase with mirror exposure.

## 4 Discussion

With this study, we aimed to test the role of sensorimotor experience in the development of facial mimicry by systematically manipulating 4‐month‐old infants’ experience with their own facial actions. Infants in the mirror condition received two weeks of daily sensorimotor experience with their own facial actions via a toy mirror, while infants in the control condition played with the same toy without the mirror for the same amount of time. Before and after this experience, we measured infants’ facial mimicry using EMG and their sensorimotor cortex activation using EEG while they observed videos of other infants’ facial actions.

We found that infants in the mirror condition showed a greater increase in sensorimotor cortex activation (specifically over the right face representation area) during the observation of other infants’ facial actions than infants in the control condition. There was also a trend towards greater mirror exposure during the two‐week training period being related to greater sensorimotor alpha suppression at post‐test. These findings suggest that short‐term, self‐generated sensorimotor experience may shape neural responses to others’ facial actions early in infancy, and are consistent with previous work by Rayson and colleagues (2017). Their study showed that infants who received greater sensorimotor experience with their own facial expressions via maternal imitation at 2 months, showed greater sensorimotor cortex activation during the observation of others’ facial expressions at 9 months. Together, these findings suggest that the amount of sensorimotor cortex activation during the observation of facial actions relates to the opportunity infants have had to form associations between the visual and motor representations of these actions either through imitative social partners or mirror self‐observation (Heyes 2001; Ray and Heyes 2011). However, the increase in activation over the right face representation area during the observation of others’ actions in the mirror group in the current study did not translate into an increase in facial mimicry as measured by EMG. Thus, although infants in the mirror group showed enhanced sensorimotor cortex activity after the two‐week training period, this may not have corresponded to activation of the specific motor representations for the facial actions they observed. This dissociation between the EEG and EMG findings suggests that experience‐related changes in sensorimotor representations may emerge earlier, or be more sensitive than, overt behavioural mimicry at this age. Below we consider several possible explanations for why increased sensorimotor cortex activation was not accompanied by measurable changes in facial mimicry in the mirror group. These explanations relate both to factors that may have limited the overall expression of facial mimicry in the sample and to factors that may have reduced differences between the mirror and control conditions.

Firstly, it could be that although infants spent a significant amount of time in front of the mirror over the two‐week training period, there may have only been relatively few instances where they would have performed frowns and smiles similar to those they observed during the pre‐ and post‐test. Instead, infants may have formed a more general association between moving their facial muscles and seeing their own face in the mirror, resulting in greater sensorimotor cortex activation at post‐test in response to seeing other infants’ faces but without an accompanying increase in activation of the corresponding facial muscles.

We chose to use a two‐week training period because we expected this time period to provide sufficient exposure for infants while also being a manageable and realistic timeframe for families to implement daily mirror exposure consistently. However, longer training periods or more targeted training approaches may be more effective. From an associative learning perspective, benefits of mirror exposure should occur even when facial actions are spontaneous rather than voluntary: spontaneous facial actions, when repeatedly paired with their visual consequences in the mirror, should strengthen the perceptual‐motor couplings that support facial mimicry. However, because infants’ facial actions may be relatively infrequent and not always performed while attending to the mirror, longer training periods or more extensive daily mirror exposure could provide more opportunities for sensorimotor learning. Infants may also have been more likely to produce positive (e.g. smiles) than negative facial expressions during the training sessions, which may have contributed to the lack of observed effects of sensorimotor training on facial mimicry overall, particularly given that mimicry scores were averaged across trial types. However, exploratory analyses (see Supplementary materials) that included trial type showed no differences in results between mouth and eyebrow actions, ruling out the possibility that effects were present only for mouth actions. Nevertheless, future interventions in which caregivers actively elicit a wider range of facial behaviours while the infant is in front of the mirror may increase sensorimotor learning opportunities beyond passive mirror exposure and could be more effective in enhancing facial mimicry. Another recommendation for future research would be to video‐record the full training sessions to allow infants’ sensorimotor experience with different facial expression to be quantified more precisely. In the current study parents were asked to upload short video clips of their infant engaging with the toy, but there was insufficient usable footage for systematic analysis.

Secondly, although caregivers in the control group were asked to restrict their infant's access to mirrors, it is likely that some infants still encountered their reflection incidentally (e.g., in car mirrors, reflective surfaces, or during video calls). These naturally occurring exposures are difficult to eliminate in home‐based designs, but may have reduced the contrast between groups in terms of self‐observation experience, potentially contributing to the absence of group differences in facial mimicry.

Thirdly, unlike our previous research in which infants viewed three repeats of each facial action per video (de Klerk et al. 2018), in the current study each action was presented only once to maximise EEG trial counts. One possibility is that the infants needed more time to process the facial actions, and would have benefitted from a less fast‐paced stimulus presentation in which each facial action was repeated several times. This is also the first study that used infant face stimuli to measure facial mimicry, and given that previous research has shown that infants generally prefer adult faces (Heron‐Delaney et al., 2017), this may have also contributed to the reduced levels of mimicry we found. However, we believe that using infant faces was an appropriate methodological choice, given that the infants observed their own reflection during the training and that from an associative learning perspective we would expect the amount of mimicry during the observation of a facial action to be proportional to the physical similarity between the observed stimulus and the stimuli that were observed when the perceptual‐motor couplings were formed (Heyes 2010). Another possible reason for the lack of evidence for facial mimicry in the present study, could be the age of our sample, as mimicry may become more robust later in the first and second year of life (e.g. Jones 2007; Kaiser et al. 2017). We chose to assess infants around 4‐months firstly, because previous research has shown evidence of facial mimicry at this age using facial EMG (e.g., de Klerk et al. 2018; Isomura and Nakano 2016), and secondly, to maximise the likelihood that the training would influence developing sensorimotor representations of facial actions. However, future interventions aiming to enhance sensorimotor experience with facial actions to increase facial mimicry may want to target older infants.

Finally, our previous research has demonstrated a relationship between maternal imitation and infant’ facial mimicry (de Klerk et al. 2019). It is therefore possible that spontaneous variation in caregiver imitation during the study period exerted a stronger influence on infants’ facial mimicry than the mirror training. In other words, if caregivers across the two conditions continued to imitate their infants’ facial actions, this may have reduced potential group differences. Future studies could manipulate caregiver imitation to test whether enhancing caregiver facial imitation leads to an increase in facial mimicry of adult female faces.

A limitation of this study is that although we preregistered repeated measures analyses, a substantial proportion of participants had missing pre‐test data—especially for the EEG analyses—which limited the feasibility of this approach without extensive imputation. To address this, we conducted an additional post‐test only analysis, which is reported as exploratory. While this analysis avoids potential biases introduced by imputation, it is statistically less powerful that the originally planned repeated measures analyses. Although the pattern of results was consistent across both analyses—demonstrating that infants in the mirror condition showed greater sensorimotor cortex activation during the observation of others infants’ facial actions—we believe this work would benefit from independent replication due to the reduced statistical power and missing data.

Another EEG‐related limitation is that we did not exclude EEG trials with overt facial movements, because we expected at least some degree of spontaneous facial responding. Supplementary analyses indicated that overt facial actions did not differ between the two conditions (see Supplementary Materials), suggesting that the observed group differences in sensorimotor alpha suppression are unlikely to be driven by systematic differences in action execution, and instead primarily reflect responses to facial action observation. However, future studies combining trial‐level facial coding with EEG could more fully disentangle observation‐related and execution‐related contributions to sensorimotor cortex activation.

Despite these limitations, the present findings show that short‐term mirror exposure enhances sensorimotor cortex activity during the observation of facial actions, without producing corresponding changes in facial mimicry. This suggests that the neural mechanisms underlying social perception can be shaped by sensorimotor experience early in infancy, and may emerge before these changes are reflected in behaviour.

## Author Contributions

**Carina C.J.M. de Klerk**: Conceptualization, investigation, methodology, formal analysis, data curation, supervision, writing – review and editing, writing – original draft, funding acquisition, project administration and visualization. **Lucy Parrett**: Resources, formal analysis, methodology, writing – review and editing and writing – original draft. **Chloe Richmond**: Formal analysis, investigation, project administration, data curation, supervision, writing – review and editin and writing – original draft. **Elizabeth Tye**: Writing – review and editing and investigation.

## Funding

This research was funded by the Fundação Bial (134/2020).

## Ethics Statement

This study was reviewed and approved by the Ethics Subcommittee of the Faculty of Science and Health at the University of Essex. All procedures were conducted in accordance with recognized ethical standards, including the Declaration of Helsinki and the UK Policy Framework for Health and Social Care Research.

## Conflicts of Interest

The authors declare no conflicts of interest.

## Declaration of AI‐assisted technologies

During the preparation of this manuscript, the authors used ChatGPT (OpenAI) to assist with language editing, and generating suggestions for analysis code used in supplementary analyses. All outputs were reviewed and edited by the authors, and the authors take full responsibility for the final content, analyses, and interpretations presented in the manuscript.

## Supporting information




**Supporting File 1**: desc70221‐sup‐0001‐SuppMat.docx

## Data Availability

The data that support the findings of this study are openly available in The University of Essex Research Data Repository at http://researchdata.essex.ac.uk/id/eprint/223.

## Endnotes

1 We preregistered that we would exclude any trials with more than 20% of the channels or 25 channels marked as bad. This was increased to 30 channels before we conducted the EEG analysis to allow us to include a more representative sample of infants and to reach the preregistered sample size within time and budgetary constraints.

2 This number was changed from 10 to 8 before we analysed the EEG data. Download the transparent changes to OSF Prereg document here: https://osf.io/v8r5y/files/osfstorage?view_only=abc123.
