# Supplementary material for: Two Weeks of Mirror Exposure Enhances Sensorimotor Cortex Activation but not Facial Mimicry in 4‐Month‐old Infants
Source: Dev Sci. 2026 May 10;29:e70221. doi: 10.1111/desc.70221 (PMC13158638; doi:10.1111/desc.70221)
Supplement: Supplementary file 1 — Supporting File 1: desc70221‐sup‐0001‐SuppMat.docx [file DESC-29-e70221-s001.docx]

**Supplementary materials**

**EMG analyses including trial type**

Because infants may have been more likely to produce positive (e.g. smiles) than negative facial expressions during the training sessions, we conducted additional exploratory EMG analyses including trial type (mouth vs. eyebrow) as an additional within-subjects factor. The results showed no significant main effect or interactions involving trial type, all *p*-values ≥ .137. The inclusion of trial type did not alter the overall pattern of results, with no significant effects of sensorimotor training on facial mimicry. These findings suggest that the absence of training effects did not result from differential responses to mouth versus eyebrow actions.

**Baseline-corrected EMG analyses**

The preregistered EMG analyses focused on standardised activation across corresponding and non-corresponding facial muscles, rather than baseline-corrected values, because valid baseline data were not available for all trials. However, to examine whether facial mimicry might be detectable when accounting for individual differences in baseline muscle activation, we conducted an additional exploratory analysis using baseline-corrected EMG data. The baseline period was defined as the 500 ms immediately preceding trial onset. For trials that were not preceded by a valid baseline (e.g. because the infant was vocalising or had something in their mouth), we used the infant’s mean baseline data across all valid baseline segments. Mimicry scores were then recalculated using these baseline-corrected values.

A repeated measures ANOVA with time (pre-test vs post-test) as the within-subjects factor and condition (mirror vs control) as the between-subjects factor, demonstrated the same pattern of results as our main preregistered analyses. There was no significant main effect of time, *F*(1, 74) = 1.814, *p* = .182, np^2^ = .024, or condition, *F*(1,74) = .324, *p* = .571, np^2^ = .004, nor an interaction between time and condition, *F*(1, 74) = .460, *p* =.500, np^2^ = .006. Additionally, we found no evidence for mimicry in either group at pre-test or post-test, all *p*-values ≥ .140 (one-sided).

**Overt facial actions**

Given concerns that overt facial movements may have influenced the EMG and EEG signal, we conducted a set of supplementary analyses examining a) the frequency of overt facial movements, and b) baseline-corrected overt facial mimicry during the stimulus presentation. These analyses assess whether group differences in overt behaviour could account for the neural effects reported in the main manuscript. These analyses were exploratory and not part of our preregistered hypotheses.

**Overt facial movements**

To examine whether the mirror group may have produced more overt facial movements, we calculated the proportion of included EMG trials in which there was any observable overt facial movement at pre- and post-test (including very subtle movements like tongue actions, slight mouth opening etc.). A repeated measures ANOVA with time (pre-test vs post-test) as within-subjects factor and condition (mirror vs control) as between-subjects factor on these scores demonstrated a significant effect of time, *F*(1,74) = 4.510, *p*=.037, driven by an overall *decrease* in the proportion of included trails with overt facial movement between pre- (M=.539), and post-test (M=.485). There was no interaction between time and condition, *F*(1,74) = 1.056, *p* = .307, nor a main effect of condition, *F*(1,74) = 0.199, *p*=.657. These analyses demonstrate that infants in the mirror and control group did not differ in how often they performed overt facial actions during stimulus presentation.

**Overt facial mimicry**

We conducted an exploratory analysis of overt (i.e. visible) facial mimicry for the infants included in the EMG analyses. Videos were coded offline and all trials in which infants performed the same facial action as the one shown on the screen were coded as 1, and trials in which they produced no action or a different action were coded as 0. Frowning was defined as a furrowing of the brow, and smiling as both corners of the mouth turning upward (with or without mouth opening). Trials in which the infant was not looking at the screen or was crying were excluded. Overt mimicry was defined as a match between the observed facial action and the infant’s corresponding action (e.g., smile for mouth trials, frown for eyebrow trials).

To account for individual differences in infants’ general tendencies to smile or frown in response to the stimuli, we calculated baseline-corrected overt mimicry scores. Baseline smiling and frowning propensities were calculated for each infant as the proportion of included stimulus trials on which they smiled or frowned, respectively. Baseline-corrected mimicry scores were then computed by subtracting each infant’s baseline propensity from their trial-level copying scores and averaging across trials. Thus positive values indicate that infants produced the corresponding actions more often than expected based on their baseline tendencies, while negative values indicate production below baseline. This approach reduces - but does not eliminate - individual variability in general expressiveness, particularly because mouth actions are typically produced more frequently than eyebrow actions at this age.

A repeated-measures ANOVA on the overt mimicry scores with time (pre-test vs post-test) and action (mouth vs eyebrow) as within-subjects factors and condition (mirror vs control) as between-subjects factor revealed no significant main effect of time, *F*(1, 74) = 1.311, *p* = .256, np^2^ = .017, and no significant effect of condition, *F*(1,74) = 2.854, *p* = .095, np^2^ = .037. There was a significant main effect of action, *F*(1, 74) = 4.424, *p*=.039, np^2^ =.056, as well as a significant interaction between time and condition, *F*(1, 74) = 4.741, *p*=.033, np^2^ =.060, and between time, action, and condition, *F*(1, 74) = 7.715, *p*=.007, np^2^ =.094.

To explore these interactions, separate repeated measures ANOVAs were conducted for each condition. In the Control condition there were no significant main effects or interactions, all *p*-values ≥ .059. In the Mirror condition, there were significant main effects of time, *F*(1, 36) = 4.543, *p* = .040, np^2^ = .112 and action, *F*(1, 36) = 4.306, *p* = .045, np^2^ = .107, and the interaction between time and action was marginally significant, *F*(1, 36) = 4.018, *p* = .053, np^2^ = .100. Together, these effects reflect overall higher levels of overt facial mimicry at pre-test compared to post-test, as well as greater overt mimicry for mouth than eyebrow actions within the Mirror condition. As illustrated in Supplementary Figure 1, this pattern was characterised by a small number of infants who showed relatively strong overt mimicry of mouth actions at pre-test. Crucially, overt facial mimicry scores at post-test – the time point at which EEG effects were observed - did not differ significantly from zero for either action type or condition with overt mimicry scores clustering near zero, all *p*-values ≥ .193. Thus despite some infants showing overt mimicry of mouth actions at pre-test, there was no evidence for overt mimicry at post-test. Taken together with the analyses of overall facial action frequency reported above, these findings suggest that it is unlikely that infants’ overt facial movements or overt facial mimicry influenced the EEG results reported in the main manuscript.

Supplementary Figure 1

*Dotplots of Mean Baseline-Corrected Overt Mimicry at Pre- and Post-test per Condition.*


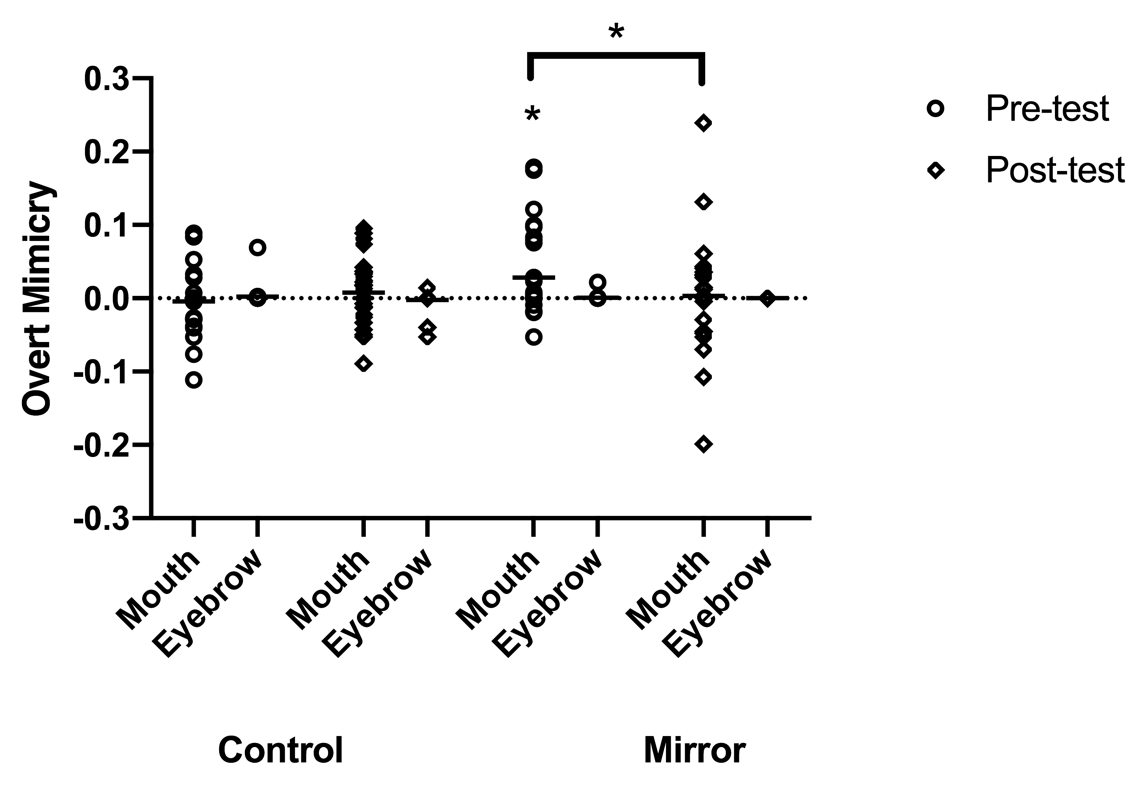


*Note.* Individual data points are marked with circles (pre-test) and diamonds (post-test). The line within each plot represents the mean. **p* < .05.
